# Morphological and histological features of thicker cartilage at the posterior medial femoral condyle in advanced knee osteoarthritis

**DOI:** 10.1016/j.ocarto.2024.100502

**Published:** 2024-07-11

**Authors:** Jeroen Geurts, François Andrey, Julien Favre, Thomas Hügle, Patrick Omoumi

**Affiliations:** a Department of Rheumatology, Lausanne University Hospital and University of Lausanne (CHUV-UNIL), Lausanne, Switzerland; b Swiss BioMotion Lab, Lausanne University Hospital and University of Lausanne (CHUV-UNIL), Lausanne, Switzerland; c Department of Radiology, Lausanne University Hospital and University of Lausanne (CHUV-UNIL), Lausanne, Switzerland

**Keywords:** Cartilage, Computed tomography, Histomorphometry, Chondrocytes, Knee

## Abstract

**Objective:**

To assess morphological and histological features of cartilage at the posterior medial condyle in advanced pre-prosthetic osteoarthritis (OA), which is notably thicker compared to non-OA knees.

**Design:**

Cartilage thickness was measured pre-operatively using MRI in 10 subjects with medial femorotibial OA (mean age: 70.2 years). Posterior condyles were obtained during arthroplasty and cartilage thickness, relative collagen content and subchondral bone volume fraction (BV/TV) were determined using phosphotungstic acid (PTA)-enhanced micro-CT. Regions of interest (ROI) around the maximum cartilage thickness were further analyzed through histomorphometry (Mankin score) and immunohistochemistry (cell density and apoptosis rates).

**Results:**

Maximum cartilage thickness was 2.63 ​± ​0.51 ​mm *in vivo* and 3.04 ​± ​0.55 ​mm *ex vivo* and both measurements were strongly correlated (*r* ​= ​0.84, *p* ​= ​0.003). Cartilaginous collagen content measured by PTA-enhanced micro-CT was negatively correlated with maximum cartilage thickness (*r* ​= ​–0.70, *p* ​= ​0.02). Average subchondral BV/TV was 31.6 ​± ​3.4% and did not correlate with cartilage thickness. Extensive loss of proteoglycan staining and tidemark multiplication were common histomorphological features around the maximum cartilage thickness. Chondrocyte densities were 315 ​± ​67 and 194 ​± ​36 ​cells/mm^2^ at the superficial and transitional cartilage zones, respectively. Chondrocyte apoptosis rates were approximately 70% in both zones. Maximum cartilage thickness correlated with superficial chondrocyte densities (*r* ​= ​0.79, *p* ​= ​0.01).

**Conclusions:**

Thicker cartilage at the posterior medial condyle in OA knees displayed degenerative changes both in cartilage tissue and at the osteochondral junction. Cartilage thickening may be influenced by alterations in the superficial zone, necessitating further investigation through molecular studies.

## 1 Introduction

Longitudinal studies on radiographic knee osteoarthritis (OA) have showed that cartilage changes may include both thinning and thickening across different medial subregions at the same time [1]. Cartilage tissue at the posterior aspect of the medial condyle is commonly preserved even in advanced stages of femorotibial OA, contrasting with the high frequency of cartilage loss in the rest of that compartment [2]. We have previously conducted cross-sectional [3] and three-dimensional imaging studies [4] to investigate cartilage thickness changes in the posterior condylar subregions compared with non-OA knees. These studies revealed that cartilage at the posterior medial condyle solely is, on average, twenty percent thicker in advanced OA compared with non-OA knees and is associated with increasing radiographic severity [3]. These imaging observations raise important questions on the anabolic mechanisms contributing to cartilage thickening in an OA joint environment.

Our understanding of the molecular mechanisms regulating cartilage thickness in OA is still limited due to the scarcity of descriptive and experimental studies on this topic. Variation of cartilage thickness across subregions in a healthy human joint seem largely independent of chondrocyte densities and matrix morphology [5]. In contrast, cartilage thickening in the context of OA may involve both changes in the cartilaginous extracellular matrix and cellular densities [[5], [6], [7], [8], [9], [10]]. Cartilage thickening through hypertrophy in experimental canine OA was associated with decreased chondrocyte densities at the superficial zones, followed by the apposition of collagen fibers [6]. Such hypertrophic cartilage repair has also been linked to sustained elevated proteoglycan production, increased chondrocyte formation and to a lesser extent, increased water content, suggesting the presence of a reparative process [6,7]. A similar transient increase of proteoglycan content has been observed in cartilage at the lateral notch of the femoral condyle in patients with an anterior cruciate ligament injury [8]. An important caveat is that these studies were conducted on weight-bearing cartilage.

In this study, we sought to characterize the morphological and histological features of cartilage tissue at the posterior aspect of the medial condyle in advanced pre-prosthetic knee OA, which is specifically thicker than in non-OA knees. We hypothesize that this analysis may inform about novel options for chondro-anabolic treatment in OA.

## 2 Materials & methods

### 2.1 Patients

Patients with advanced femorotibial OA (*n* ​= ​10; mean age ​= ​70.2 ​± ​6.7 years; 5 female) scheduled to undergo total knee joint arthroplasty were included in this prospective study. Exclusions criteria were rheumatic disorders and a history of joint injury (post-traumatic OA). Written informed consent was obtained from all participants. The study procedures followed were in accordance with the ethical standards of the local ethics committee (CER-VD) and with the Helsinki Declaration of 1975, as revised in 2000.

### 2.2 MRI

MRI was performed the day prior to the knee joint arthroplasty. MR images were acquired on a 3.0T scanner (Siemens 3T Magnetom Prisma Fit, Siemens Healthcare, Erlangen, Germany). The acquisition parameters included 2D fat-suppressed intermediate-weighted sequences (35 ​ms) in sagittal, coronal and axial planes intermediate-weighted sequences as well as 3D fat suppressed intermediate-weighted and double-echo steady-state sequences. All acquired data were systematically archived on a picture archiving and communication system workstation (Carestream Client version 13; Carestream Health, Rochester, NY, USA).

### 2.3 Image analysis

**Fig. 1:**
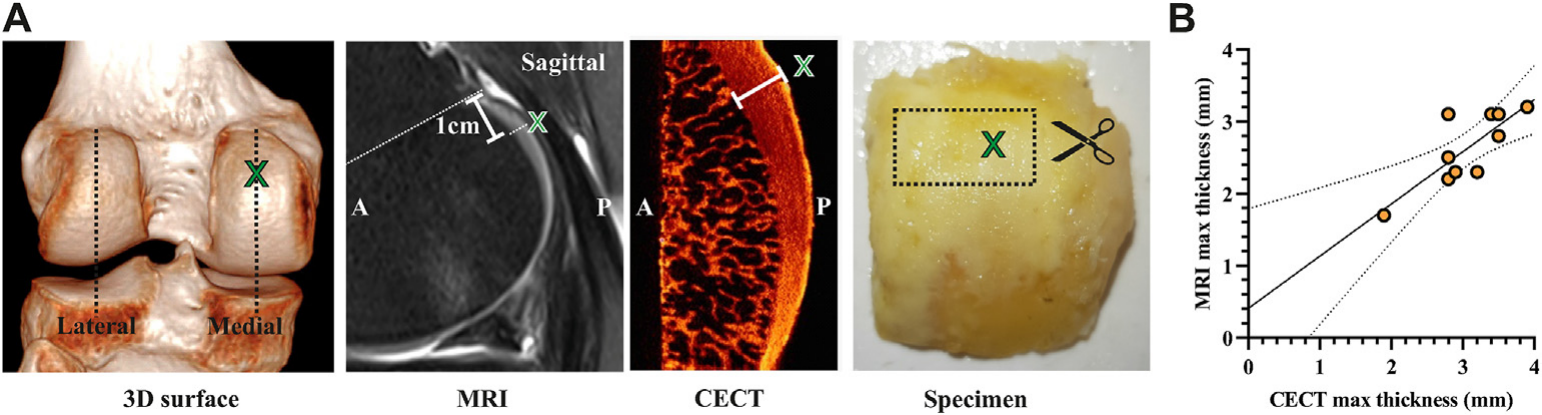
**In vivo and ex vivo measurement of cartilage thickness at the non-weight bearing posterior medial femoral condyle. (A)** 3D surface-rendering reformat of a representative CT image illustrating the planes of interest through the midportion of the posterior condyles. Cartilage thickness was measured at 1 ​cm from the physeal line by MRI. Postoperative maximum cartilage thickness was measured in resected posterior condyles by contrast enhanced microCT (CECT). The areas surrounding the maximum cartilage thickness (green X) were dissected and processed for histological analyses. A ​= ​anterior, P ​= ​posterior. **(B)** Scatter plot of maximum cartilage thickness measured *in vivo* and *ex vivo* using MRI and CECT, respectively. Regression line with 95% confidence intervals is depicted. (For interpretation of the references to color in this figure legend, the reader is referred to the Web version of this article).

Cartilage thickness at the posterior aspect of the medial femoral condyle was measured with a digital caliper on two sagittal multiplanar reformats from 3D sequences through the midportion of each condyle (Fig. 1A). Briefly, the sagittal plane of each femoral condyle was defined, in reference to the axial plane, as the plane passing through the midportion of each condyle and perpendicular to the posterior subchondral bone plate. A zoom factor of 2.5 was used. Cartilage thickness at the center of the half most medial portion of posterior aspect of femoral condyles [4] was measured (rounded off to the nearest tenth of a mm) on a line perpendicular to the subchondral bone plate, between the subchondral bone plate and the surface of the cartilage, 10 ​mm from the physeal line.

### 2.4 Contrast-enhanced micro-computed tomography scanning

Articular cartilage was labeled with phosphotungstic acid (PTA) using the method developed by Nieminen *et al*. [11]. Posterior medial and lateral condyles were harvested during joint arthroplasty and fixed in 10% neutral buffered formalin. After two rinses in saline for 1 ​h, specimens were immersed in 1% (w/v) PTA pH 2.71 in 70% (v/v) ethanol for 14 days at room temperature. Subsequently, samples were briefly rinsed in 70% ethanol before micro-computed tomography (μCT) imaging. Specimens were scanned using a benchtop μCT scanner (Skyscan 1076, Bruker, Kontich, Belgium) using the following parameters: 50 ​kV, 200 ​μA, 300 ​ms exposure time, 1° rotation step, 35 ​μm resolution, 0.5 ​mm aluminium filter. Acquired X-ray projections were reconstructed using Skyscan NRecon (V1.7.0.4, Bruker) and default corrections for beam hardening and ring artefacts were applied.

### 2.5 PTA-enhanced μCT image analysis

**Fig. 2:**
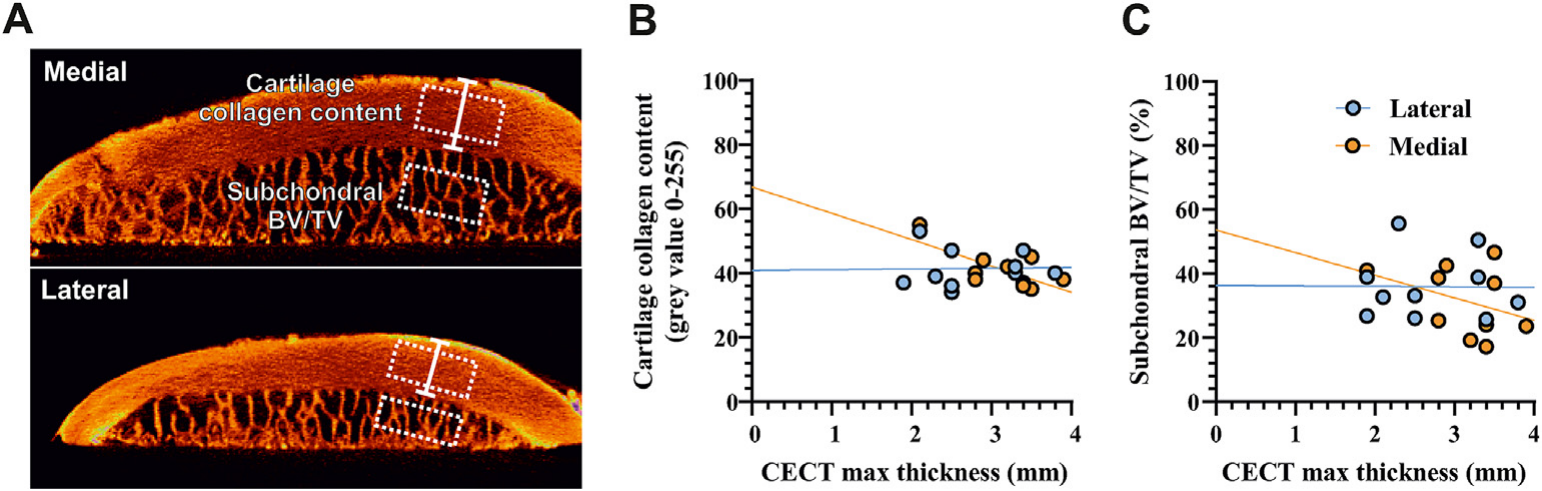
**Increased cartilage thickness at the posterior medial condyle associates with lower collagen content (A)** Regions-of-interest used for measurements of subchondral trabecular bone volume fraction (BV/TV) and cartilage collagen content near the maximal cartilage thickness (white arrow). Representative sagittal CECT images of medial and lateral posterior condyles are shown **(B,C)** Scatter plot of maximum cartilage thickness *ex vivo* versus cartilage collagen content and subchondral BV/TV. Regression lines are depicted for posterior medial (orange) and lateral condyles (blue). (For interpretation of the references to color in this figure legend, the reader is referred to the Web version of this article).

The subregion with the thickest cartilage was identified in coronal and sagittal planes using 3D visualization software (DataViewer V1.5.3.4, Bruker). Maximum cartilage thickness was measured on a line perpendicular to the subchondral cortical plate (Fig. 1A). CT-analyser software (CTAn, V1.16.9.0, Bruker) was used to measure relative cartilaginous collagen content and subchondral trabecular bone volume fraction (BV/TV) within 3.5 ​× ​3.5 ​mm ROIs around the maximum cartilage thickness (Fig. 2A). ROIs in articular cartilage were positioned at 0.5 ​mm from surfaces of the cartilage and subchondral cortical plate and average gray value of the volume was used as a measure PTA staining intensity. Subchondral trabecular BV/TV was determined on binary thresholded images within a Regions of interest located at 0.5 ​mm from the tidemark.

### 2.6 Histology and immunohistochemistry

Specimens were destained by immersion in PTA washout buffer (0.1 ​M Na_2_HPO_4_, 137 ​mM NaCl, 2.7 ​mM KCL, 0.55 ​mM NaOH, pH 10) for 7 days at room temperature [12]. Subsequently, samples were decalcified in 5% formic acid and the subregion around the maximum cartilage thickness was dissected and embedded in paraffin. Tissue sections were stained with Safranin-*O* & Fast Green and cartilage degeneration was graded using Mankin score. Chondrocytes were counted in superficial and transitional zones of cartilage (upper 20% of cartilage thickness [5]) and normalized for tissue area. Chondrocyte apoptosis was determined using an Apoptag kit according to the manufacturer's instructions (ApopTag Plus Peroxidase *In Situ* Apoptosis Kit, Merck, Darmstadt, Germany). Total and Apoptag-labeled cells were counted by two independent observers.

### 2.7 Statistical analysis

This study was based on a convenience sample and no formal power calculations were performed. Numerical data followed a normal distribution. Mankin scores were treated as ordinal data. Correlation analyses were performed using Pearson (numerical data) or Spearman (Mankin score) correlation. Simple linear regression analyses were performed for graphical presentation of correlations. Within-patient comparison of chondrocyte densities was performed using ratio paired *t*-test. *P*-values of less than 0.05 were considered significant. Data were analyzed and plotted using GraphPad Prism 9.3 (GraphPad Software, Inc, La Jolla, USA).

## 3 Results

### 3.1 Cartilage thickness at the posterior medial condyle associates with reduced cartilaginous collagen content

Maximum cartilage thickness *in vivo* at the posterior aspect of the medial condyle was 2.63 ​± ​0.51 ​mm (range 1.7–3.1 ​mm). Maximum cartilage thickness *ex vivo* of resected posterior medial condyles was 3.04 ​± ​0.55 ​mm (range 1.9–3.9 ​mm). Cartilage thickness measurements *in vivo* and *ex vivo* were strongly correlated (Pearson *r* ​= ​0.84, *p* ​= ​0.0025) and *ex vivo* imaging confirmed the location of maximum cartilage thickness at the center of the half most medial portion of the posterior medial condyle, as reported previously [4] (Fig. 1).

The relative collagen content, measured as X-ray attenuation by the collagen-binding contrast agent, was negatively correlated with cartilage thickness in the posterior medial condyle (Pearson *r* ​= ​−0.70, *p* ​= ​0.023) (Fig. 2B). Subchondral BV/TV was not significantly correlated with cartilage thickness (Pearson *r* ​= ​−0.36, *p* ​= ​0.29) (Fig. 2C). Cartilage thickness in lateral condyles was not correlated with collagen content (Pearson *r* ​= ​0.02, *p* ​= ​0.94) or subchondral BV/TV (Pearson *r* ​= ​−0.01, *p* ​= ​0.98), which further justifies our particular focus on medial posterior condyles.

### 3.2 Morphological and histological features of cartilage at the posterior medial condyle

**Fig. 3:**
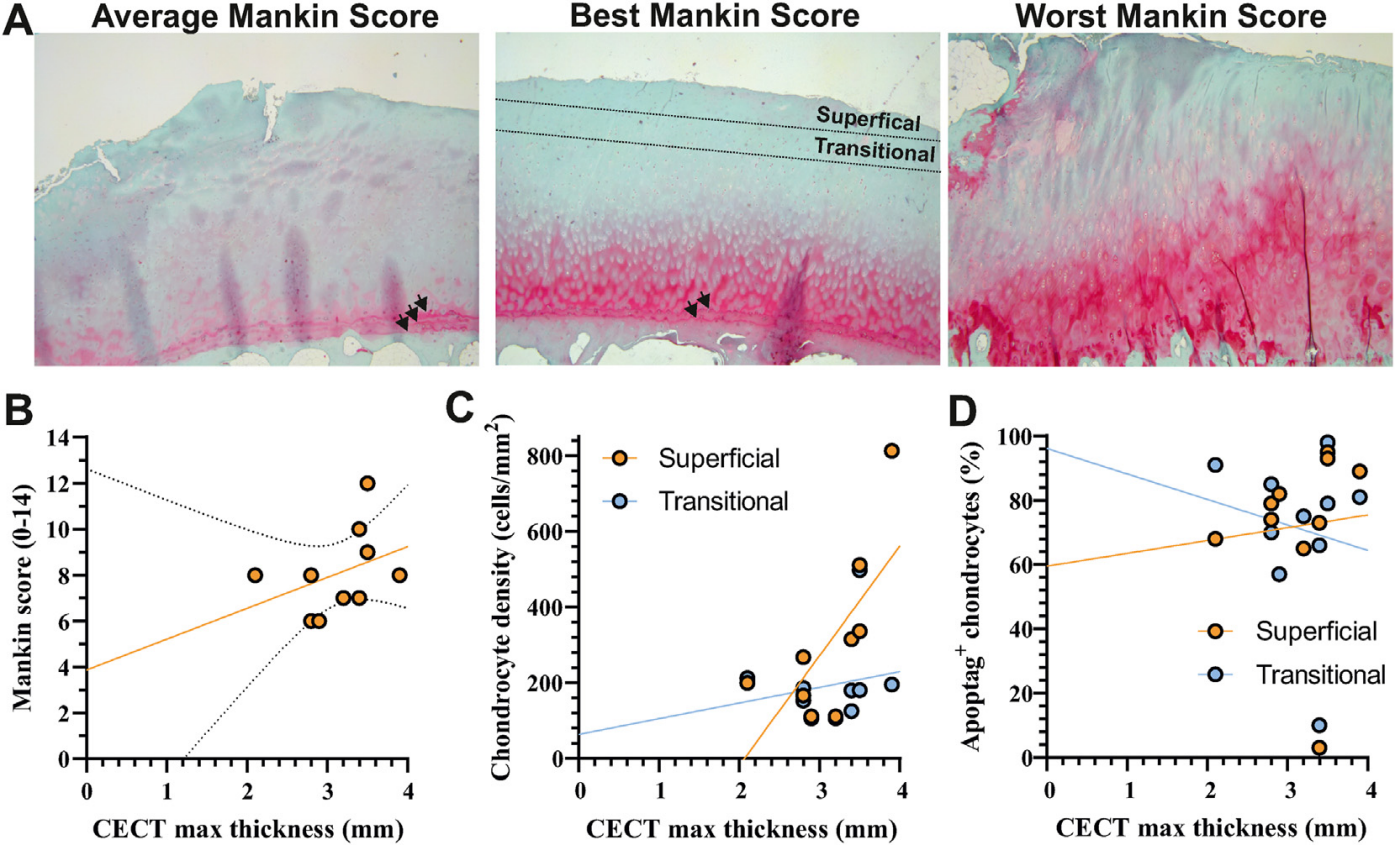
**Cartilage at posterior medial condyles displays histopathological features of OA (A)** Representative images displaying cartilage with the average (8 - left panel), lowest (6 - middle panel) and highest (12, right panel) Mankin score. Tidemark duplication was a common feature at posterior medial condyles (arrowheads). Superficial and transitional cartilage zone are indicated. **(B**–**D)** Scatter plot of maximum cartilage thickness *ex vivo* versus Mankin score, chondrocyte density and Apoptag-positive cells. Superficial and transitional zones of cartilage were defined as the ten percent volume fraction beneath the articular surface and superficial zone, respectively. Regression lines are depicted for superficial and transitional zones.

Histopathological features of OA including loss of proteoglycan staining and tidemark multiplication were observed in all medial posterior condyles (Fig. 3A). Structural damage at the articular cartilage was low or absent and chondrocyte cloning was not detected.

The median Mankin score was 8 (range 6–12) and did not correlate with cartilage thickness (Spearman *r* ​= ​0.50, *p* ​= ​0.14) (Fig. 3B). Chondrocyte densities were 315 ​± ​213 and 194 ​± ​113 ​cells/mm^2^ at the superficial and transitional zones (upper 20% of cartilage thickness) and higher in superficial zones (*p =* 0.03, ratio paired-*t*-test). Chondrocyte densities in the superficial zone were correlated with cartilage thickness (Pearson *r* ​= ​0.79, *p* ​= ​0.01) (Fig. 3C). There were no differences in apoptag-positive chondrocytes between superficial (72.1 ​± ​26.3%) and transitional (71.2 ​± ​24.6%) zones. Apoptosis rates in superficial (Pearson *r* ​= ​0.07, *p* ​= ​0.83) and transitional zones (Pearson *r* ​= ​−0.16, *p* ​= ​0.65) were not correlated with cartilage thickness (Fig. 3D).

## 4 Discussion

This study sought to describe morphological and histological features of cartilage at the posterior medial condyle in OA knees, which was found to be thicker (2.63 ​± ​0.51 ​mm) compared with non-OA knees (2.14 ​± ​0.36 ​mm [4]). Contrary to our initial hypothesis, cartilage thickening at this location did not necessarily denote healthy cartilage. Cartilage thickness was associated with decreased collagen content and increased chondrocyte densities in the superficial zone of the cartilage. While cartilage thickening at this non-weight-bearing subregion may be driven by biochemical rather than biomechanical responses at the cartilage surface, more nuanced molecular analyses are required to further elucidate chondro-anabolic mechanisms.

Previous studies on weight-bearing human and canine cartilage have suggested that cartilage thickening may be mechanistically linked to a transient increase in proteoglycan and water content in response to injury-induced changes to joint biomechanics [[6], [7], [8]]. However, this mechanism seems less likely for posterior medial condyles due to the excessive loss of proteoglycan staining observed in all samples. Release of growth factors, such as connective tissue growth factor, from the pericellular matrix of cartilage and other joint tissues is known to regulate cartilage responses to loading and injury [9]. Interestingly, CTGF-null mice displayed paradoxical cartilage thickening, presumably caused by compensatory regulation of transforming growth factor-beta (TGF-beta) ligands from non-cartilaginous joint tissues. We hypothesize that similar paracrine mechanisms may be involved in regulating chondro-anabolic effects in human OA. In support of this, cartilage thickening at the external femoral condyle was associated with osteophyte formation in the same subregion in the absence of joint space narrowing, suggesting a chondro-regenerative joint microenvironment in the early stages of OA [13].

The fact that cartilage thickening is predominantly observed at the posterior aspect of the medial rather than the lateral condyle is likely explained by the kinematics of the knee in high flexion angles, as previously reported [2,14]. From a cellular point of view, Quinn *et al*. described that differences in superficial zone morphology, including higher chondrocyte cell densities, were most pronounced at the medial femoral subregion [5]. Chondrocyte densities at superficial (96 ​± ​13/mm^2^) and transitional (85 ​± ​6/mm^2^) zones in non-OA femoral condyles were significant lower than observed in this study, suggesting that cartilage thickening may have resulted from chondrocyte proliferation in the superficial zone. As a limitation, we did not perform analysis of proliferation markers in cartilage and apoptosis rates were similar between superficial and transitional zones. It remains to be elucidated whether subregional variations in cartilage can give rise to differential responses to joint microenvironmental factors.

Another possible explanation for cartilage thickening could be an expansion of the calcified cartilage layer without affecting the non-calcified cartilage. Although we found tidemark multiplication in the most specimens, the advancement of calcified cartilage typically covered distances below 100 ​μm. Transient unloading or immobilization of the knee joint has been described to accelerate tidemark advancement [15]. In this context, our observation of tidemark duplication may be due to the non-weight-bearing nature of the posterior condyle rather than a specific remodeling response to OA.

Several limitations should be considered when interpreting the results. The cross-sectional design of the study did not allow us to investigate whether chondrocyte densities change over time. Additionally, reference values for chondrocyte densities in age- and sex-matched non-OA knee joints remain unavailable at present. Longitudinal studies on knees advancing to joint arthroplasty, such as the OA initiative, may provide conclusive evidence that cartilage at the posterior medial condyle thickens during disease progression. Future research should pursue more nuanced molecular analyses, such as comparative transcriptomics of chondrocytes in different cartilage layers or subregions, to leverage the findings on cartilage thickening for the development of novel treatment options.

In conclusion, our study provides insights into the morphological and histological characteristics of cartilage at the posterior medial condyle in OA knees and its association with altered collagen content and chondrocyte densities. While our findings suggest potential involvement of biochemical mechanisms in cartilage thickening, further research utilizing nuanced molecular analyses is warranted to fully elucidate chondro-anabolic mechanisms in an OA environment.

## Author contributions

Conception and design: Omoumi, Favre, Hügle, Geurts.

Provision of study material: Omoumi, Favre.

Radiological assessment: Omoumi, Favre.

Analysis and interpretation of data: Omoumi, Favre, Andrey, Hügle, Geurts.

Drafting of the article: Omoumi, Favre, Andrey, Hügle, Geurts.

Final approval of the article: Omoumi, Favre, Andrey, Hügle, Geurts.

## Role of the funding source

The funders had no role in study design, data collection and analysis or drafting and submission of the manuscript.

## Declaration of competing interest

The authors declare that they have no conflicts of interest related to this study.
